# Relationship Between Preoperative Shoulder Osteoarthritis Severity Score and Postoperative PROMIS-UE Score After Rotator Cuff Repair

**DOI:** 10.1177/23259671221143801

**Published:** 2023-01-04

**Authors:** Michael R. Davies, Natalie Kucirek, Daria Motamedi, C. Benjamin Ma, Brian T. Feeley, Drew Lansdown

**Affiliations:** * Department of Orthopaedic Surgery, University of California, San Francisco, San Francisco, California, USA.; *Investigation performed at University of California, San Francisco, California, USA.*

**Keywords:** SOAS score, shoulder MRI, rotator cuff repair

## Abstract

**Background:**

Mild to moderate glenohumeral joint osteoarthritis is a common finding among patients who are evaluated for rotator cuff tears. However, the impact of preoperative shoulder joint degeneration on patient-reported outcomes after rotator cuff repair (RCR) is not well-established.

**Purpose:**

To apply the magnetic resonance imaging (MRI)–based Shoulder Osteoarthritis Severity (SOAS) score to the evaluation of patients undergoing RCR and determine the relationship between preoperative shoulder pathology present on MRI and postoperative Patient-Reported Outcomes Measurement Information System–Upper Extremity (PROMIS-UE) scores.

**Study Design:**

Case-control study; Level of evidence, 3.

**Methods:**

Seventy-one MRI scans corresponding to 71 patients were analyzed by 2 independent reviewers and scored using the SOAS criteria. Intraclass correlation coefficients were calculated for total SOAS score as well as for each subscore. Spearman correlations were calculated between averaged SOAS scores, patient characteristics, and PROMIS-UE scores. Linear regression analysis was performed between the independent variables of patient age, sex, body mass index, and significant SOAS score components determined by univariate analysis with the dependent variable of PROMIS-UE score. Significance was defined as *P* < .05 for univariate analyses and < .0125 for multivariate analyses using the Bonferroni correction.

**Results:**

The mean PROMIS-UE score of this cohort was 51.5 ± 7.4, while the mean total SOAS score was 21.5 ± 8.4. There was a negative correlation between total SOAS score and postoperative PROMIS-UE score (*r* = –0.24; *P* = .040). Both cartilage wear (*r* = –0.33; *P* = .0045) and acromioclavicular joint degeneration (*r* = –0.24; *P* = .048) individually demonstrated negative correlations with PROMIS-UE score. When a multivariate linear regression with Bonferroni correction was applied to the significant variables identified in univariate analysis along with patient characteristics, none were independently correlated with PROMIS-UE score.

**Conclusion:**

In this cohort of patients undergoing RCR, increasing preoperative total SOAS score was predictive of lower postoperative PROMIS-UE scores. SOAS subscores with the strongest negative correlations with PROMIS-UE scores included cartilage wear and acromioclavicular joint degeneration. The cartilage subscore was negatively correlated with PROMIS-UE scores independent of patient factors in multivariate analysis.

Rotator cuff repair (RCR) is a common orthopaedic procedure, with an estimated 250,000 to 300,000 repairs performed annually in the United States.^12,28^ The incidence of RCR has increased steadily over the past 2 decades, likely in part due to the rise of arthroscopy, enhanced diagnostic imaging, and an aging population.^2,5^ Given the growing number of patients undergoing RCR, numerous studies have sought to characterize the risk factors that contribute to inferior postoperative outcomes. Variables associated with worse patient-reported outcomes (PROs) after RCR include female sex,^8,20^ high degree of fatty infiltration,^6,20,22^ smoking,^4,25^ increased body mass index (BMI),^1,4^ older age,^22^ workers’ compensation claim,^4,8,10^ and larger tear size.^18,22^

The presence of preoperative glenohumeral joint osteoarthritis (GHOA) may also affect RCR outcomes, although the existing literature on this association is limited and conflicting. Two studies reported that patients with radiographically diagnosed GHOA preoperatively had worse postoperative PRO scores after RCR compared with those without preoperative GHOA.^15,17^ In contrast, other studies found no difference in postoperative PROs between those with and without preoperative GHOA.^13^ A recent study by Reddy et al^24^ found no difference in revisions, retear rates, or PROs in patients with GHOA undergoing cuff repair compared with those without GHOA. A sizable subset of patients undergoing RCR may have preoperatively detectable GHOA, with reported rates ranging from 12.9% to 28% in the literature.^15,17,19,21^ Most studies rely on radiographic scoring systems to diagnose GHOA, including the Samilson-Prieto, Kellgren-Lawrence, and Guyette systems. While these scoring systems have high inter- and intraobserver reliability, they classify GHOA based on joint space narrowing, osteophyte presence, and sclerosis,^26^ which may be more difficult to detect in early disease.

Therefore, magnetic resonance imaging (MRI) has been suggested as an alternative imaging technique to assess shoulder OA. MRI better detects the subtle changes in cartilage, alterations in soft tissue, and presence of joint inflammation that may be seen in early OA.^11^ In 2019, Jungmann et al^14^ introduced a new, MRI-based classification system for quantifying shoulder OA known as the Shoulder Osteoarthritis Severity (SOAS) score. The SOAS score is a semiquantitative metric assessing global severity of OA in the shoulder joint. It comprises 6 subcategories, including rotator cuff, labral-bicipital complex, cartilage, osseous findings, joint capsule, and acromion, with the overall score totaling 0 to 100. The SOAS score correlated strongly with existing radiographic Kellgren-Lawrence and Samilson scores and demonstrated an interobserver reliability of 0.96 to 0.98.^14^

In this study, we sought to evaluate the association between preoperative shoulder joint degeneration, as assessed by the MRI-based SOAS score, and postoperative outcomes in patients undergoing RCR. Given that RCR preserves the shoulder joint, we hypothesized that patients with higher SOAS scores, and therefore more significant joint pathology before surgery, would have lower PROs after RCR. This hypothesis was also informed by prior studies reporting GHOA as a risk factor for poorer outcomes after RCR.^15,16,17^

## Methods

### Study Design and Participants

We conducted a retrospective cohort study using a prospectively collected database of patients with RCR from a single tertiary referral center. All patients completed the Patient-Reported Outcomes Measurement Information System–Upper Extremity (PROMIS-UE) form postoperatively after RCR.^23^ We included patients who underwent RCR and had preoperative shoulder MRI scans (n = 84) with a minimum of 15 months (range, 17-70 months) of follow-up after surgery. Exclusion criteria were patients with inadequate MRI studies (n = 6), RCR performed for acute shoulder dislocation (n = 5), revision RCR (n = 1), and open RCR (n = 1). This left us with 71 shoulders from 71 patients that were included in the analyses. All surgeries were performed by 1 of 3 fellowship-trained surgeons (C.B.M., B.T.F., D.L.). MRI scans were assessed by 2 independent reviewers (M.R.D., D.M.) and graded using the SOAS score as described by Jungmann et al^14^ (see Appendix Table A1).

### Study Variables

Patient variables of age, sex, and BMI were recorded. The PROMIS-UE form was utilized to assess postoperative outcomes. PROMIS-UE is the upper extremity subset of PROMIS, a computerized system developed by the US National Institutes of Health to standardize and streamline PRO reporting. The PROMIS-UE has been validated for rotator cuff injury and studied to establish substantial clinical benefit and Patient Acceptable Symptom State) values after RCR.^9^ PROMIS-UE scores have also been shown to improve over the course of RCR recovery^7^ and correlate with legacy PROs, including the American Shoulder and Elbow Surgeons Standardized Shoulder Assessment Form and Simple Shoulder Test, in patients undergoing RCR.^23^

Standardized preoperative MRI scans were collected within 1 year before RCR surgery. SOAS scores were calculated by the same 2 independent reviewers using the classification specifications described by Jungmann et al.^14^ These include scoring of supraspinatus, infraspinatus, and teres minor tear size; subscapularis tear size; rotator cuff retraction; fatty infiltration; muscle atrophy; glenoid labrum; paralabral ganglia; long head of biceps tendon; glenohumeral ligaments; cartilage quality; bone marrow edema; intraosseous cysts; osteophytes; bone deformity; synovitis; joint effusion; loose bodies; degree of bursitis; acromioclavicular (AC) joint degeneration; and acromion deformity. These scores were summed to produce an SOAS score from 0 to 100, with a higher score representing more severe degenerative changes of the shoulder joint. An average measurement between the 2 reviewers was utilized for subsequent correlational and regression analysis.

### Statistical Analysis

Statistical analyses were performed in Stata (Version 16.1; StataCorp LP). Descriptive statistics including mean and standard deviation were calculated. Intraclass correlation coefficients (ICCs) were calculated between reviewers for both total SOAS score and each individual criterion. Individual SOAS subscores that were not found to have a significant ICC were not included in further analyses. The Spearman rank correlation coefficient (*r*) was used to assess relationships between pairs of continuous variables, including total SOAS scores and patient variables, total SOAS scores and PROMIS-UE scores, and individual SOAS criterion scores and PROMIS-UE scores. The point biserial correlation was used to compare sex with PROMIS-UE and SOAS scores. A multivariate linear regression was performed with the independent variables of patient age, sex, BMI, and total SOAS score or subscore and the dependent variable of PROMIS-UE score, with *P* < .0125 considered significant after application of the Bonferroni correction with m = 4 comparisons. Statistical significance was defined as *P* < .05 for all other tests.

## Results

A total of 71 patients were included for MRI scoring and subsequent analysis. The average follow-up was 42.9 ± 12.1 months (range, 17-70 months). The average patient age was 62.4 years, with an average BMI of 27.2 and a mean PROMIS-UE score of 51.5 ± 7.4 (Table 1). Two patients underwent concomitant AC joint surgery at the time of RCR consistent with partial distal clavicle resection. Over the follow-up period, 2 patients experienced rotator cuff retear and 1 patient subsequently underwent reverse total shoulder arthroplasty. Comparison of SOAS scores between the 2 reviewers demonstrated an overall ICC of 0.63 (Table 2).

**Table 1:** Characteristics of Study Patients*^a^*

Variable	Value
Age, y	62.4 ± 7.7 (39-77)
Sex	
Male	43
Female	28
Body mass index	27.2 ± 7.3 (18.53-35.73)
PROMIS-UE score	51.5 ± 7.4 (25.9-56.4)
Follow-up, mo	42.9 ± 12.1 (17-70)
AC joint surgery	2
Cuff retear	2

*^a^* Data are reported as mean ± SD (range) or No. of patients. AC, acromioclavicular; PROMIS-UE, Patient-Reported Outcomes Measurement Information System–Upper Extremity.

**Table 2:** Interreviewer ICCs and Mean SOAS Scores*^a^*

MRI Parameter	ICC (*P* < .05)	SOAS Score (Mean ± SD)
Total SOAS score	0.63	21.48 ± 8.41
Rotator cuff		
Supra-/infraspinatus tear size	0.47	3.50 ± 1.03
Subscapularis tear size	0.57	1.96 ± 1.01
Retraction	0.53	1.61 ± 0.99
Fatty infiltration	0.36	1.45 ± 1.20
Atrophy	0.70	1.43 ± 1.46
Labral-bicipital complex		
Labrum	0.66	1.76 ± 1.05
Long head of biceps	0.68	1.18 ± 0.72
Paralabral ganglia	ns	0.18 ± 0.40
GH ligaments	0.38	0.41 ± 0.58
Cartilage		
GH articular cartilage	0.27	1.96 ± 1.82
Osseous findings		
Bone marrow edema	0.22	0.15 ± 0.37
Subchondral cysts	ns	0.58 ± 0.54
Osteophytes	ns	0.33 ± 0.57
Bone deformity	ns	0.049 ± 0.15
Joint capsule		
Synovitis	ns	0.63 ± 0.49
Joint effusion	0.51	0.64 ± 0.69
Loose bodies	0.42	0.085 ± 0.29
Acromion		
Subacromial bursitis	0.22	1.18 ± 0.55
AC joint degeneration	0.57	1.96 ± 0.67
Acromion deformity	ns	0.099 ± 0.28

*^a^* AC, acromioclavicular; GH, glenohumeral; ICC, intraclass correlation coefficient; MRI, magnetic resonance imaging; ns, not significant; SOAS, Shoulder Osteoarthritis Severity.

The average total SOAS score for this cohort was 21.48 ± 8.41 (Table 2). This represents mild arthritis, as Jungmann et al^14^ found a score of 32 and above to represent manifest OA corresponding to a Kellgren-Lawrence grade ≥2. The average scores for individual subscores are listed in Table 2. In this cohort, rotator cuff subscores contributed the most points to the total SOAS score, with the highest individual component score coming from supraspinatus and infraspinatus tears (Table 2). The subcategory with the lowest average contribution to total SOAS score was “Osseous findings,” corresponding with the lowest ICCs between reviewers, as these were overall rare findings that were mild in severity in this cohort of patients.

SOAS scores were averaged between reviewers and correlated with patient data and PROMIS-UE scores (Table 3). We found a significant positive correlation between patient age and total SOAS score (*r* = 0.49; *P* < .001). There were no other significant correlations between patient data and either total SOAS score or PROMIS-UE score. There was a significant negative correlation between total SOAS scores and PROMIS-UE scores (*r* = –0.24; *P* = .040). Among SOAS subscores, we found that the cartilage (*r* = –0.33; *P* = .0045) and AC joint degeneration (*r* = –0.24; *P* = .048) score were negatively correlated with PROMIS-UE scores (Table 3).

**Table 3:** Summary of Correlations*^a^*

Comparison	*r*	*P*
Patient characteristics		
Total SOAS vs age	0.49	**<.001**
PROMIS-UE vs age	–0.068	.58
Total SOAS vs sex	–0.17	.16
PROMIS-UE vs sex	–0.013	.91
Total SOAS vs BMI	0.13	.28
PROMIS-UE vs BMI	–0.16	.18
Total SOAS score		
Total SOAS vs PROMIS-UE	–0.24	**.040**
Rotator cuff		
Supra-/infraspinatus tear size vs PROMIS-UE	–0.19	.11
Subscapularis tear size vs PROMIS-UE	–0.13	.28
Retraction vs PROMIS-UE	–0.090	.45
Fatty infiltration vs PROMIS-UE	0.11	.36
Atrophy vs PROMIS-UE	–0.16	.18
Labrum/biceps		
Labrum vs PROMIS-UE	–0.099	.41
Long head of biceps vs PROMIS-UE	–0.12	.33
GH ligaments vs PROMIS-UE	0.070	.56
Cartilage		
Cartilage vs PROMIS-UE	–0.33	**.0045**
Osseous findings		
Bone marrow edema vs PROMIS-UE	–0.12	.30
Joint capsule		
Joint effusion vs PROMIS-UE	–0.084	.49
Loose bodies vs PROMIS-UE	–0.033	.78
Acromion		
Subacromial bursitis vs PROMIS-UE	–0.18	.14
AC joint degeneration vs PROMIS-UE	–0.24	**.048**

*^a^* Boldface *P* values indicate statistical significance (*P* < .05). AC, acromioclavicular; BMI, body mass index; GH, glenohumeral; PROMIS-UE, Patient-Reported Outcomes Measurement Information System–Upper Extremity; SOAS, Shoulder Osteoarthritis Severity.

Given the moderate positive correlation between total SOAS score and patient age, we next sought to determine whether the total SOAS score as well as cartilage and AC joint degeneration scores were associated with PROMIS-UE scores independent of patient age, sex, and BMI. We found that in a multivariate linear regression with a Bonferroni correction, neither total SOAS score nor the cartilage or AC joint subscore was independently associated with PROMIS-UE scores independent of patient characteristics (Table 4).

**Table 4:** Multivariate Regression of SOAS Variables to Predict PROMIS-UE*^a^*

	β Coefficient	SE	*P^b^*
Age	0.062	0.13	.64
Sex (female = 0, male = 1)	–0.68	1.83	.71
BMI	–0.047	0.12	.71
Total SOAS	–0.22	0.13	.081
Age	–0.012	0.12	.92
Sex (female = 0, male = 1)	–0.63	1.8	.73
BMI	–0.059	0.12	.63
Cartilage	–1.05	0.50	.038
Age	0.015	0.12	.91
Sex (female = 0, male = 1)	0.61	1.89	.75
BMI	–0.046	0.12	.71
AC degeneration	–2.41	1.48	.11

*^a^* AC, acromioclavicular; BMI, body mass index; PROMIS-UE, Patient-Reported Outcomes Measurement Information System–Upper Extremity; SOAS, Shoulder Osteoarthritis Severity.

*^b^* The threshold for significance was *P* < .0125 after applying the Bonferroni correction.

## Discussion

In this study, we have applied the MRI-based SOAS score to a retrospectively obtained cohort of patients who underwent RCR and determined that increasing preoperative SOAS score was negatively correlated with postoperative PROMIS-UE scores at an average of 43 months after surgery. Analyzing the individual components of the SOAS score, we found that cartilage degeneration and advanced AC joint degeneration were each negatively correlated with PROMIS-UE scores in univariate but not multivariate analysis with patient characteristics. The results of this study suggest that increasing degenerative pathology of the shoulder joint on MRI before RCR is associated with lower PROs after surgery.

Prior studies have established a number of predictors of RCR failure, which in turn have been linked with lower PROs after surgery. Wylie et al^29^ found that patients who underwent RCR with successful tendon healing reported higher PROs and that MRI-based risk factors for lack of tendon healing included tear size, retraction, and fatty infiltration. In our study, we found that while individually each of these cuff-related variables was not significantly associated with PROMIS-UE scores at follow-up, collectively the total SOAS score demonstrated a significant weak correlation with PROMIS-UE scores. This suggests that overall glenohumeral joint pathology may be more predictive of outcomes after RCR than any particular characteristic of rotator cuff tears. This finding is especially important to consider when counseling patients regarding surgical treatment options. Future studies may look to clarify if there is an objective, MRI-based threshold of glenohumeral joint degeneration at which reverse shoulder replacement may be preferred over RCR.

When analyzing the individual SOAS score components, we found that increasing glenohumeral cartilage degeneration demonstrated a negative correlation with PROMIS-UE score. Prior studies using radiographic classifications for GHOA have demonstrated mixed results when assessing the impact of OA on RCR outcomes. Jeong et al^13^ reported no significant difference in Constant scores between patients undergoing RCR with and without radiographic evidence of OA graded using the modified Samilson and Prieto classification. In contrast, several other studies have reported lower PROs in patients with radiographically confirmed GHOA at the time of RCR.^15,16^ In our study, the overall rates and severity of GHOA were low on preoperative MRI, yet patients with increased cartilage wear demonstrated lower postoperative PROMIS-UE scores. This negative correlation was significant, while rotator cuff–specific components of the SOAS score, including tear size and atrophy, demonstrated weaker nonsignificant negative associations with the PROMIS-UE score. Thus, in this cohort of patients with relatively preserved glenohumeral joints and a range of rotator cuff pathology, outcomes may be influenced more by even mild cartilage wear present at the time of surgery than by the classically described rotator cuff parameters. However, this finding must be interpreted in the context of the low ICC found for the cartilage subscore.

We additionally found that increasing AC joint pathology on MRI correlated with worse postoperative PROMIS-UE scores. In this cohort, only 2 patients underwent AC joint surgery concomitantly with RCR. The overall rates of acromial deformity were low among this cohort of patients. However, AC joint degeneration of at least mild to moderate severity was a common finding and contributed more points on average to the total SOAS score than either rotator cuff tendon retraction, rotator cuff muscle fatty infiltration, or muscle atrophy (Table 3). Prior research has assessed the association between AC joint radiographic degeneration and rotator cuff tears, finding that while AC joint morphological variations do not correlate with rotator cuff tears, increasing degenerative findings of the AC joint are predictive of cuff tears.^3^ Several studies have assessed the impact of treating AC joint arthritis concurrently with rotator cuff disease, with the overall finding that distal clavicle resection to treat AC joint arthritis did not improve functional outcome scores among patients with RCR.^27^ In light of these prior studies and the evidence that we present in this study, we posit that severe AC joint degeneration may indicate globally advanced shoulder degeneration rather than an isolated therapeutic target for improving outcomes of RCR. However, further studies are needed to reexamine the role of AC joint degeneration in the outcomes after RCR given the findings presented here.

### Limitations

This study has several inherent limitations. This study is a retrospective evaluation, and we lack preoperative PROMIS-UE scores to allow for determination of magnitude of improvement from baseline. The observations, however, are novel and are worth further study in a prospective fashion. Additionally, the ICCs for total SOAS score and its subcomponents ranged from moderate for the total score to weak for certain individual subscores. The SOAS score was designed to comprehensively assess a wide range of glenohumeral joint pathology, with scores ranging from 0 to 100, while the overall pathology in this cohort ranged from mild to moderate. We therefore feel that the reported reliability of scores in this study represents a realistic clinical application of this scoring system, rather than an ideal application to a broader range of joint pathology as originally described by Jungmann et al.^14^ Similarly, the significant correlations between SOAS parameters and PROMIS-UE scores uncovered by this study remained weak to moderate in magnitude. This observation indicates that glenohumeral joint degeneration may be responsible for a portion of the observed outcome score, although muscle quality, patient characteristics, physical therapy compliance, and other unmeasured factors certainly contribute to outcomes as well. Finally, the Bonferroni correction method was applied for multiple comparisons in the multivariate analysis, which increases the chance of a type 2 error in this setting. Future prospective studies can clarify the interrelationship of these numerous confounding factors on eventual outcomes.

## Conclusion

In this study, we demonstrated that increasing overall preoperative shoulder joint pathology measured using the MRI-based SOAS score negatively correlated with PROMIS-UE scores after RCR surgery. We found that the most prominent contributors to this association were increasing cartilage wear and increasing AC joint degeneration in univariate analysis, although these factors were not significant in multivariate analysis with patient characteristics. Taken together, factors beyond rotator cuff tear size and muscle quality, including overall joint degeneration on preoperative MRI, should be taken into consideration and discussed with patients being evaluated for RCR.

## APPENDIX

**Appendix Table A1:** SOAS Scoring Method*^a^*

1. Rotator cuff (0-35 points) ▪ Tear size (SS, IS, TM), Bateman: 0-6 points ▪ Tear size (SSC), Fox and Romeo: 0-5 points ▪ Retraction (any tendon), Patte: 0-3 points ▪ Muscle fatty infiltration (individual scores), Goutallier: 0-4 points ▪ Muscle atrophy, Thomazeau: 0-3 points
2. Labral-bicipital complex ▪ Glenoid labrum (anterior and posterior assessed separately): 0-2 points ▪ Paralabral ganglia: 0-3 points ▪ Long head of biceps tendinopathy: 0-3 points ▪ Glenohumeral ligaments (any ligament): 0-2 points
3. Cartilage ▪ Glenoid/humerus scored separately: 0-12 points
4. Osseous findings ▪ Bone marrow edema: 0-3 points ▪ Intraosseous cysts: 0-3 points ▪ Osteophytes: 0-3 points ▪ Bone deformity: 0-3 points
5. Joint capsule ▪ Synovitis/obliteration of joint capsule (rotator interval and axillary recess assessed separately): 0-2 points ▪ Joint effusion: 0-3 points ▪ Loose bodies: 0-2 points
6. Acromion ▪ Bursa: 0-3 points ▪ AC joint degeneration: 0-3 points ▪ Acromion deformity: 0-2 points

*^a^* The total Shoulder Osteoarthritis Severity (SOAS) score ranges from 0 to 100 points. AC, acromioclavicular; IS, infraspinatus; SS, supraspinatus; SSC, subscapularis; TM, teres minor.
